# Casp8 acts through A20 to inhibit PD‐L1 expression: The mechanism and its implication in immunotherapy

**DOI:** 10.1111/cas.14932

**Published:** 2021-05-20

**Authors:** Jiahuan Zou, Hongwei Xia, Chenliang Zhang, Huanji Xu, Qiulin Tang, Gongmin Zhu, Jielang Li, Feng Bi

**Affiliations:** ^1^ Department of Abdominal Oncology Cancer Center West China Hospital Sichuan University Chengdu China; ^2^ Laboratory of Molecular Targeted Therapy in Oncology West China Hospital Sichuan University Chengdu China

**Keywords:** A20, Casp8, immunotherapy, PD‐L1, posttranslational modification

## Abstract

Immunotherapy targeting the PD‐L1/PD‐1 pathway is a novel type of clinical cancer treatment, but only small subsets of patients can benefit from it because of multiple factors. PD‐L1/PD‐1 expression is a biomarker for predicting the efficacy of anti‐PD‐L1/PD‐1 therapy, which highlights the importance of understanding the regulatory mechanisms of PD‐L1 expression in cancer cells. Casp8 is an apical caspase protease involved in mediating cell apoptosis, but it also has multiple nonapoptotic functions. Casp8 mutations are associated with increased risks of cancer, and low expression of Casp8 is closely connected with poor prognosis in patients with cancer. In addition, mutations of Casp8 in lymphocytes also lead to human immunodeficiency, thereby causing dysfunction of the innate immune system, but the roles of Casp8 in antitumor immunity remain unclear. Here, we found that knocking down Casp8 in mouse melanoma cells promoted tumor progression in an immune system–dependent manner. Mechanistically, Casp8 induced PD‐L1 degradation by upregulating TNFAIP3 (A20) expression, a ubiquitin‐editing enzyme that results in PD‐L1 ubiquitination. In addition, compared with Casp8^fl/fl^ mice, mice with conditional deletion of Casp8 in natural killer (NK) cells (Ncr1^iCre/+^Casp8^fl/fl^ mice) showed a decreased frequency of IFN‐γ+ and CD107a+ NK cells but an increased frequency of PD‐1+ and CTLA‐4+ NK cells. Melanoma cells with Casp8 knocked down exhibited sensitivity to anti‐PD‐1 or anti‐CTLA‐4 antibody treatments, particularly in Ncr1iCre/+Casp8fl/fl mice. Together, the results indicate that Casp8 induces PD‐L1 degradation by upregulating A20 expression and that decreased Casp8 expression is a potential biomarker for predicting the sensitivity to anti‐PD‐L1/PD‐1 immunotherapy.

## Graphical abstract

Casp8 induces PD‐L1 degradation by upregulating A20 expression, and decreased Casp8 expression is a potential biomarker for predicting the sensitivity to anti‐PD‐L1/PD‐1 immunotherapy.

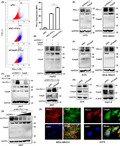

## 1 INTRODUCTION

The programmed cell death‐ligand (PD‐L) 1/programmed cell death protein (PD)‐1 pathway is a critical immune checkpoint and has become a major focus of research in antitumor immunotherapy.^1^ Cancer cells escape immune surveillance by suppressing the proliferation of immune cells, as well as blocking their infiltration and cytokine release.^2^ A major characteristic of tumor immune evasion is the expression of inhibitory ligands, such as PD‐L1, on the surface of cancer cells.^3^ The PD‐L1 protein is expressed not only on tumor cells but also on the surface of immune cells, including T cells, DC cells, macrophages, and NK cells.^4^, ^5^, ^6^ When binding to PD‐1, PD‐L1 inhibits cytotoxic T lymphocyte (CTL) activation and induces apoptosis or functional exhaustion of CTLs.^3^, ^7^ Antibody‐based drugs such as nivolumab or atezolizumab have been developed to block the interaction of PD‐L1 and PD‐1 with the intention of recovering the function of CTLs, and these efforts have led to great success with multiple cancer treatments.^8^, ^9^ The clinical benefits suggest that it is necessary to study the regulatory mechanisms of PD‐L1 expression. Previous studies reported that PD‐L1 can be regulated at the transcriptional level by JAK/STAT, c‐Myc, etc.^10^, ^11^, ^12^ PD‐L1 posttranslational modification (PTM), such as protein ubiquitination, glycosylation, phosphorylation, and palmitoylation, has also emerged as a critical mechanism of resistance to PD‐L1/PD‐1 blockade therapy.^13^, ^14^, ^15^

Casp8 is an aspartate‐specific cysteine protease that acts as an initiator caspase in extrinsic cell death pathways induced by tumor necrosis factor (TNF) family members.^16^, ^17^ Pro‐Casp8 is recruited and activated via reciprocal cleavage to form the death‐inducing signaling complex (DISC) by binding with the adaptor protein FADD. Then the activated Casp8 initiates the downstream apoptotic cascade by cleaving Casp3, Casp7, or Bid.^18^, ^19^, ^20^ Recent studies have indicated that Casp8 can act as molecular switch for apoptosis, necroptosis, and pyroptosis, which play critical roles in organ development and tissue homeostasis, and knocking out Casp8 or enzymatically inactivating Casp8 (C362A) in mice causes embryonic lethality.^21^ Casp8 is specifically overexpressed in lymphocytes, including T cells, B cells, and NK cells. Germline point mutation of Casp8 results in pleiotropic defects in lymphocyte activation and leads to human immunodeficiency.^22^, ^23^ Patients with inflammatory bowel disease (IBD) with inherited Casp8 deficiency also exhibit T cell and B cell dysfunction.^24^, ^25^, ^26^ Moreover, several studies have reported that multiple cancers carry different Casp8 mutants; for example, Casp8 undergoes somatic frameshift mutations in hepatocellular carcinomas and colorectal carcinomas and point mutations in breast cancer.^27^, ^28^, ^29^ These mutations are deemed to be associated with increased cancer risk. These studies have suggested that Casp8 plays critical roles in the inflammatory process and innate immunity, but the functions of Casp8 in antitumor immunity and immunotherapy remain unclear.

In our present study, we found that overexpression of Casp8 induced ubiquitination of PD‐L1, while knocking down A20, a ubiquitin‐editing enzyme, attenuated this ubiquitination. Knockdown of Casp8 in melanoma cells accelerates tumor growth in an immune‐dependent manner. Moreover, using flow cytometry, we analyzed the PBMCs of patients with advanced cancer, and the results suggested that the population of Casp8^‐^ NK cells (NK cells with low Casp8 expression) had a lower ability to produce IFN‐γ and CD107a than Casp8^+^ NK cells (NK cells with high Casp8 expression), but Casp8^‐^ NK cells had a higher expression of PD‐1 and CTLA‐4. We also identified that conditional knockout of Casp8 in NK cells caused a reduction in IFN‐γ and CD107a secretion and an increase in PD‐1 and CTLA‐4 expression, thereby promoting tumor growth of melanoma cells in Ncr1^iCre/+^Casp8^fl/fl^ mice. Next, we validated that the negative effect of Casp8 can be alleviated by treatment with anti‐PD‐1 antibody or anti‐CTLA‐4 antibody, and mice with low Casp8 expression showed better sensitivity to monoclonal antibody (mAb, anti‐PD‐1 antibody and anti‐CTLA‐4 antibody) treatments. These results indicated that the expression level of Casp8 in cancer may be an indicator for evaluating the sensitivity of patients to mAb therapy.

## 2 MATERIALS AND METHODS

### 2.1 Antibodies and reagents

Antibodies and reagents used in the study are listed in Tables S1 and S2.

### 2.2 Cell culture and treatment

All cell lines (HepG2, HCT‐116, HEK‐293T, MDA‐MB231, A375, B16, 4T1, Hep1‐6) were obtained from the Cell Bank of Type Culture Collection of Chinese Academy of Science, Shanghai, China, with mycoplasma contamination detection and STR profiling. All cancer cells were maintained with DMEM supplemented with 10% fetal bovine serum at 37ºC with 5% CO2.

### 2.3 Western blot and immunoprecipitation

The cells were harvested and washed with ice‐cold PBS twice; then, the cells were resuspended in lysis buffer (containing 1% protease inhibitor and 1% phosphatase inhibitor) on ice for 30 minutes and centrifuged at 15 700 *g* for 30 minutes at 4°C. The protein concentration was measured quantitatively by the Pierce BCA Protein Assay Kit. Part of the supernatant was taken as input, and the remaining part was incubated with 2 μg indicated antibodies overnight and then incubated with 20 μl Protein A/G Magnetic Beads for 2 hours at 4°C. After washing with lysis buffer five times, the immunoprecipitation samples were boiled with 1× loading buffer for immunoblotting analysis.

The immunoprecipitation and input samples were subjected to electrophoresis by 10% SDS‐PAGE; then, the protein was transferred onto a polyvinylidene difluoride membrane. The membranes were blocked with 5% bovine serum albumin (BSA) for 1 hour and incubated with indicated primary antibodies overnight at 4°C. The membranes were washed with Tris‐HCl buffered saline contained 0.1% Tween‐20 (TBST) three times and incubated with HRP‐labeled secondary antibodies for 1 hour at room temperature (RT). UltraSignal hypersensitive ECL substrate was used to detect the HRP. Visualized images were obtained from a chemiluminescence imaging system (Fusion FX). The percentage of intensity of the protein bands was quantified using ImageJ 1.53a software. The experiment was carried out in triplicate and repeated at least twice.

### 2.4 Reverse transcription PCR (RT‑PCR)

The samples were subjected to total RNA extraction using the Animal Total RNA Isolation Kit (Fore Gene Co. Ltd.) according to the manufacturer's protocol. The total RNA was reversely transcribed into cDNA by a reverse transcription system (TAKARA). Quantitative PCR was performed using 2× SYBR Green qPCR Mix (Biomake) in a real‐time PCR machine (CFX Connect Real‐time System, BioRad). The primer sequences are presented in Table S3. The experiment was carried out in triplicate and repeated at least twice.

### 2.5 Small interfering RNA (siRNA) and plasmids

The siRNA sequences are presented in Table S4. The efficiency of siRNAs was detected by Western blot and RT‐PCR.

The plasmid pcDNA3.1‐Casp8‐flag and vector were obtained from Public Protein/Plasmid Library (BC028223); pCMV‐PD‐L1‐His, pCMV‐PD‐L1‐GFP, and pCMV‐A20‐Flag were purchased from Sino Biological Inc (HG10084‐CH, HG10084‐ACG, HG12089‐NF); and pcDNA3.1‐ubiquitin‐HA and pCMV‐EGFP were obtained from Dr Zhang. All plasmids were confirmed by DNA sequencing (TSING KE Biological Technology).

### 2.6 Double immunofluorescence staining

The cells were seeded into round coverslips and treated as indicated. After washing twice with PBS, the cells were fixed with 3% paraformaldehyde for 10 minutes at 37℃ and permeabilized with 0.1% BriJ 98 for 90 seconds at RT. The samples were blocked with 10% goat serum for 1 hour at RT and incubate with anti‐PD‐L1 primary antibodies overnight at 4℃. Next, the coverslips were washed with PBS three times and incubated with goat anti‐rabbit IgG‐Alexa Fluor® Plus 647 secondary antibody for 2 hours at RT. The samples were blocked again and incubated with anti‐Casp8 or anti‐A20 antibodies for 3 hours at RT, followed by goat anti‐mouse IgG‐Alexa Fluor® 488 secondary antibody after washing three times. Nuclear staining was performed by DAPI. A confocal microscope (N‐STORM & A1) was used for image analysis.

### 2.7 Bioinformatics analysis

The raw data of cancer patients were downloaded from The Cancer Genome Atlas (TCGA, http://cancergenome.nih.gov). R Studio software was used to perform Kaplan‐Meier survival analyses.

### 2.8 Flow cytometry

The function of NK cells in the peripheral blood of patients and spleens of mice was also detected by flow cytometry. A total of 20 mL red blood cell lysis buffer was added into 2‐mL blood samples and mixed gently. The mixtures were left at RT for 10 minutes and then centrifuged at 400 g for 10 minutes to get the cell precipitation. The samples were washed with PBS twice and stimulated with ionomycin (1 μg/mL) and PMA (50 ng/mL) for 4 hours. Brefeldin A was used to inhibit protein transport from the endoplasmic reticulum to the Golgi apparatus. The samples were incubated with CD16/CD32 antibody to block FcγR binding for 15 minutes. The molecules expressed on cytomembranes were stained by antibodies for 30 minutes at 4°C. Next the cells were fixed/permeabilized with the BD Cytofix/Cytoperm™ Fixation/Permeabilization Solution Kit according to the manufacturer's protocol and subjected to staining of intracellular molecules. For mice spleens, the samples were ground and filtered with a 70 μm mesh. The following experimental steps were the same as above. All samples were subjected to flow cytometry analysis by using FACSSymphony (BD Biosciences). The data were processed using Flowjo Vx.07.

### 2.9 Site‐directed mutagenesis assay

A single‐point mutation C360S was carried out to get the Casp8‐C360S plasmid without enzymatic activity based on the full‐length Casp8 plasmid, while the double‐point mutations Y614A and F615A were performed to get A20‐ZnF4 mutation or C779A and C782A to get A20‐ZnF7 mutation based on the full‐length A20 plasmid. All mutations were confirmed by DNA sequencing (TSING KE Biological Technology).

### 2.10 Generation of stable Casp8 knockdown cells using lentiviral infection

The lentiviral‐based shRNA used to knock down the expression of Casp8 in mice cell lines was obtained from TSING KE Biological Technology. The shRNA target sequence is presented in Table S4.

### 2.11 Mouse tumorigenesis and immunotherapy assays

B16/shNC or B16/shCasp8 cells (1.5 × 10^5^) were resuspended in 100 µL of PBS and injected subcutaneously (s.c.) into the right flank of 5‐week‐old male nude mice or immunocompetent C57BL/6J mice. The mice were monitored every 2 days after injection. Tumor size was recorded by calipers, and the tumor volumes were calculated. The tumor weights were measured after sacrifice on the 17th day after injection.

B16shNC or B16/shCasp8 cells (2 × 10^5^) were resuspended in 100 µL of PBS and inoculated s.c. into Casp8^fl/fl^ mice or Ncr1^iCre/+^Casp8^fl/fl^ mice as design. The mice were injected intraperitoneally (i.p.) with 100 μg of anti‐PD‐1 antibody, anti‐CTLA‐4 antibody, or PBS every 3 days for four times in total. Tumor size of mice was recorded. Tumor weights were also measured after sacrifice on the 21st day after treatment. Tumor tissues resected from mice were subjected to Western blotting to detect PD‐L1, A20, and granzyme B expression. ImageJ 1.53a software was used to quantify the density of the protein bands.

### 2.12 Animal breeding and care

Nude mice and C57BL/6 mice were purchased from Beijing HFK Bioscience Co, Ltd. The Casp8^fl/fl^ mice were purchased from Jackson Laboratory (027 002) and the B6/J^Nju‐Ncr1em1Cin(iCre)/Nju^(T005674) mice were obtained from Nanjing Biomedical Research Institute of Nanjing University.

### 2.13 Statistical analysis

GraphPad Prism software (version 6.02) was used to carry out the statistical analysis of all the data. Two‐tailed Student's *t*‐test was used to assess the difference between two experimental groups. Significance was determined as a *P‐*value <.05 (*), <.01 (**), and <.001 (***). Differences that were nonsignificant were denoted by ns.

## 3 RESULTS

### 3.1 Casp8 downregulates PD‐L1 protein expression

**FIGURE 1:**
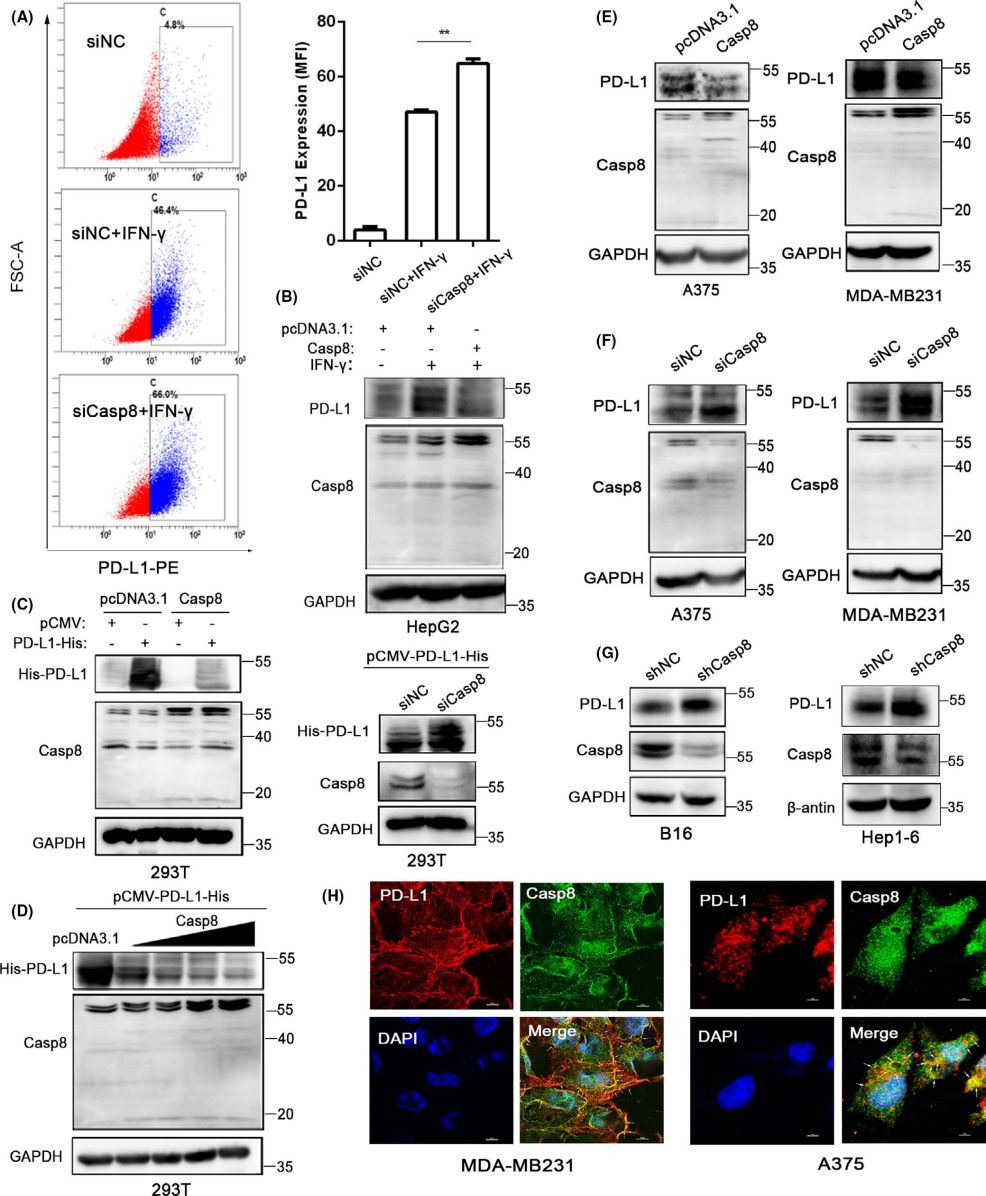
Casp8 downregulates PD‐L1 protein expression. A, HepG2 cells were transfected with siNC or siCasp8 for 24 h and stimulated with IFN‐γ (50 ng/mL) for 24 h. Induced PD‐L1 expression on the cytomembrane was quantified by flow cytometry. The experiment was carried out in triplicate and repeated at least twice. The data show the means ± SD, *P‐*value<.01 (**). B, Western blot analysis of IFN‐γ–induced PD‐L1 expression in the HepG2 cells transfected with Casp8 plasmids for 48 h. C, Western blot analysis of PD‐L1 expression in 293T cells expressing His‐PD‐L1 and transfected with Casp8 plasmids or siCasp8. Exogenous His‐PD‐L1 in cells was detected by the anti‐6xHis antibody. D, Western blot analysis of PD‐L1 expression in 293T cells expressing His‐PD‐L1 and transfected with Casp8 plasmids at different concentration gradients (0.5 µg, 1 µg, 1.5 µg, and 2 µg). E, F, Western blot analysis of the expression of intrinsic PD‐L1 in A375 and MDA‐MB‐231 cells after transfection with Casp8 plasmids (E) or siCasp8 (F) for 24 h or 48 h. G, Hep1‐6 and B16 cells were infected with shNC or shCasp8, and the expression of PD‐L1 and Casp8 was detected by Western blot analysis. H, Confocal microscopy image showing the protein expression of PD‐L1 and Casp8 in MDA‐MB231 and A375 cells (indicated by white arrows). Scale bar: 10 µm for the MDA‐MB231 cells and 5 µm for the A375 cells

PD‐L1 protein expression can be classified into constitutive expression and inducible expression in epithelial cells and squamous cells.^30^ We detected intrinsic PD‐L1 expression in various cell lines by RT‐PCR, and the results showed that HepG2, Bel‐7402, and HCT‐116 cells expressed lower PD‐L1 levels than A375 and MDA‐MB231 cells (Figure S1A). Interferon‐γ (IFN‐γ) is the most potent stimulator of the inducible expression of PD‐L1. Gene silencing experiments were performed with siRNAs, and PD‐L1 expression induced by IFN‐γ at the protein level was determined by flow cytometry. The results showed that knocking down Casp8 expression using si RNAs increased IFN‐γ–induced PD‐L1 expression in HepG2 cells (Figure 1A). The efficacy of Casp8 siRNAs was assessed by quantitative RT‐PCR (Figure S1B). To verify these results, HepG2, HCT116, and Bel‐7402 cells were transfected with Casp8 plasmids in the presence of IFN‐γ, and the results from Western blot assays showed that the overexpression of Casp8 downregulated the PD‐L1 expression induced by IFN‐γ at the protein level (Figure 1B, Figure S1C). Moreover, we also measured PD‐L1 expression at the mRNA level by RT‐PCR, and we found that transfection with Casp8 siRNAs or Casp8 plasmids had no effect on PD‐L1 expression at the transcriptional level (Figure S1D). Next, we cotransfected 293T cells with exogenous His‐PD‐L1 plasmids and Casp8 plasmids or siCasp8, and the changes in PD‐L1 protein expression were consistent with the results described above (Figure 1C). We also found that the degradation of PD‐L1 caused by Casp8 was dose‐dependent (Figure 1D). To validate the correlation of Casp8 and intrinsic expression of PD‐L1, A375 and MDA‐MB231 cells, which have intrinsically high PD‐L1 expression, were transfected with Casp8 plasmids, and Western blot assays were performed to measure PD‐L1 expression. The results suggested that overexpression of Casp8 downregulated intrinsic PD‐L1 protein expression (Figure 1E). Next, we reduced Casp8 expression with siRNAs and examined the changes in PD‐L1 expression. The Western blot assays showed that knocking down Casp8 upregulated PD‐L1 expression (Figure 1F). Similar results were obtained from mouse B16 and Hep‐1‐6 cells infected with shCasp8 (Figure 1G). To better delineate the correlation of Casp8 and PD‐L1, we sought to determine whether PD‐L1 colocalized with Casp8. Colocalization of PD‐L1 and Casp8 was confirmed in MDA‐MB231 and A375 cells by immunofluorescence double staining, through which the PD‐L1 protein was visually confirmed to colocalize with the Casp8 protein (Figure 1H). All these results indicate that Casp8 can interact with PD‐L1 and that overexpression of Casp8 decreases intrinsic and extrinsic PD‐L1 expression.

### 3.2 Activated Casp8 induces ubiquitination of the PD‐L1 protein

**FIGURE 2:**
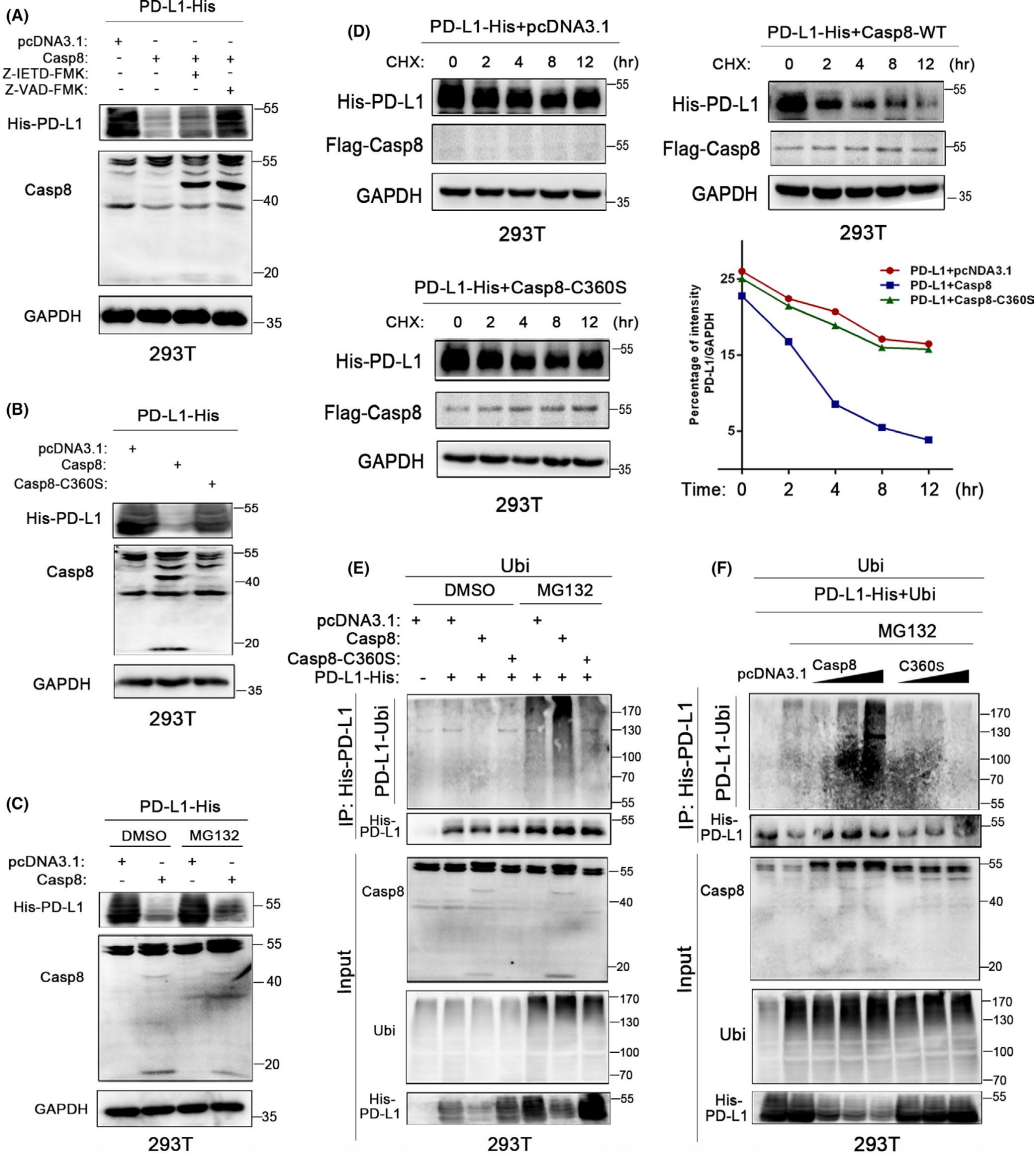
Activated Casp8 induces ubiquitination of the PD‐L1 protein. A, 293T cells expressing His‐PD‐L1 were transfected with vector or Casp8 plasmids in the presence of Z‐IETD‐FMK (50 µmol/L) or Z‐VAD‐FMK (10 µmol/L) for 24 h, and exogenous His‐PD‐L1 in the cells was detected by the anti‐6xHis antibody. B, Western blot analysis of PD‐L1 expression in 293T cells expressing His‐PD‐L1 and transfected with pcDNA3.1, Casp8‐WT, or Casp8‐C360S mutation. C, Western blot analysis of His‐PD‐L1 expression in 293T cells expressing His‐PD‐L1, transfected with Flag‐Casp8, and treated with MG132 (3 µmol/L) for 24 h. D, Protein stability of His‐PD‐L1 in 293T cells. The cells were transfected with pcDNA3.1, Casp8‐WT, or Casp8‐C360S and treated with cycloheximide (25 µg/mL). Western blot analysis of His‐PD‐L1 expression at the indicated times. E, Ubiquitination assay of His‐PD‐L1 in 293T cells. The cells were transfected with various plasmids as described and treated with DMSO or MG132 (3 µmol/L) for 24 h. Ubiquitinated PD‐L1 was pulled down by anti‐6xHis tag antibody, and Western blotting was performed with anti‐ubiquitin antibody. F, 293T cells were transfected with the indicated plasmids of different concentration gradients and treated with MG132 (3 µmol/L). Ubiquitinated PD‐L1 was immunoprecipitated by anti‐6xHis antibody and detected by Western blot assay

An increasing number of studies have reported that enzymatic activity is required for Casp8 function.^18^, ^19^, ^20^ To validate whether Casp8‐induced PD‐L1 degradation is related to caspase enzymatic activity, Z‐IETD‐FMK and Z‐VAD‐FMK were used to inhibit the enzymatic activity of Casp8 and total caspases. His‐PD‐L1–expressing 293T cells were transfected with Casp8 plasmids in the presence of Z‐IETD‐FMK or Z‐VAD‐FMK for 24 hours. Western blot results suggested that the degradation of PD‐L1 caused by Casp8 can be attenuated by the inhibitors (Figure 2A). Site‐directed mutagenesis was performed to construct enzymatically inactive Casp8 plasmids (Casp8‐C360S). Western blot assays showed that the Casp8 promotion of PD‐L1 degradation was abolished in cells transfected with the Casp8‐C360S mutant (Figure 2B). Subsequently, we examined whether Casp8 affected PD‐L1 degradation through proteolysis. Altered PD‐L1 levels in cells with Casp8 overexpression were restored upon incubation with MG132 (Figure 2C). A half‐life analysis using cycloheximide also suggested that activated Casp8 accelerated PD‐L1 degradation (Figure 2D). To further dissect these dynamics, we sought to determine whether activated Casp8 increased PD‐L1 ubiquitination. The results suggested that overexpression of Casp8‐WT, but not the Casp8‐C360S mutant, induced an increase in PD‐L1 ubiquitination in a dose‐dependent manner (Figure 2E and F, Figure S2A‐B). In conclusion, all these results demonstrated that Casp8 enzyme activity was required for Casp8‐induced PD‐L1 ubiquitination.

### 3.3 Casp8 upregulates A20 expression to promote PD‐L1 degradation

**FIGURE 3:**
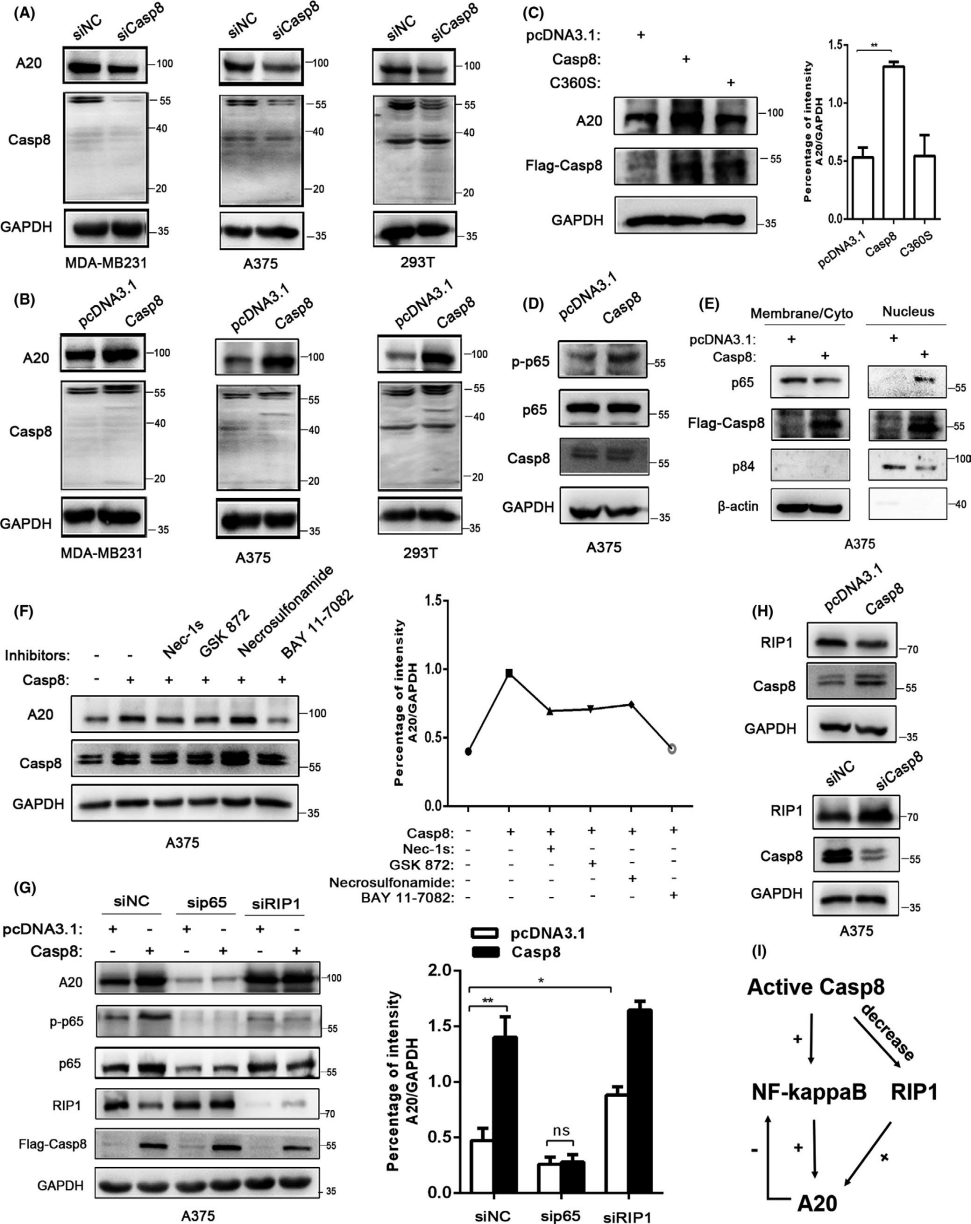
Casp8 increases A20 expression through NF‐kappaB and RIP1. A, B, 293T, MDA‐MB231, and A375 cells were transfected with siCasp8 for 48 h (A) or Casp8 plasmids for 24 h (B), and Western blotting was used to detect the expression of A20. C, Western blot analysis of A20 expression in A375 cells transfected with Casp8 or the Casp8‐C360S mutant for 24 h. ImageJ was used to analyze the gray lanes. Histogram indicated the intensity of A20/GAPDH. Student's *t* test, *P‐*value <.01 (**). D, Western blot analysis of p‐p65 expression in A375 cells transfected with Casp8 for 24 h. E, A375 cells were transfected with pcDNA3.1 or Flag‐Casp8 plasmids for 24 h, and the nuclear translocation of p65 was detected. F, A20 expression in A375‐overexpressing Casp8 cells in the presence of Nec‐1s (10 µmol/L), GSK872 (10 µmol/L), necrosulfonamide (10 µmol/L), and BAY‐11‐7082 (5 µmol/L) for 24 h was measured by Western blotting. G, A375 cells were transfected with sip65 or siRIP1 for 24 h and then with Casp8 plasmids for 24 h. Western blotting was performed to measure A20 expression. ImageJ was used to analyze the gray lanes. Histogram indicated the intensity of A20/GAPDH. *P* <.05 (*), <.01 (**), unpaired two‐tailed *t*‐test. H, Western blot analysis of RIP1 expression in A375 cells transfected with Casp8 plasmids or siCasp8 for 24 h or 48 h. I, The mechanisms of active Casp8‐regulated A20 expression

Casp8 not only plays a role in apoptosis and necrosis but can also act as a scaffold protein to promote the production of cytokines.^16^, ^17^, ^31^ However, there is currently no evidence that Casp8 acts as an E3 ubiquitin ligase. We hypothesized that Casp8 may not interact with PD‐L1 directly but regulates PD‐L1 degradation by upregulating a currently unknown E3‐ubiquitin ligase. Previous studies have shown that A20, a bifunctional ubiquitin‐editing enzyme, impedes the polyubiquitination of Casp8,^32^ but whether activated Casp8 has negative feedback regulation on A20 expression is unclear. In our studies, gene silencing experiments were performed with siRNAs, and the results suggested that knocking down Casp8 downregulated the expression of A20 (Figure 3A, Figure S3A). Next, we measured the expression of A20 in 293T, A375, MDA‐MB231, and murine B16 cells after transfection with Casp8 plasmids. Western blot analysis showed that overexpression of activated Casp8 upregulated A20 expression (Figure 3B, Figure S3B). Moreover, the enzymatically inactive Casp8‐C360S mutant did not affect A20 expression in A375 cells (Figure 3C). Previous studies have shown that Casp8 is required for the activation of NF‐kappaB (Figure 3D), which contributes to the transcriptional regulation of A20^33^, ^34^ (Figure S3C). Here, we confirmed that Casp8 could accelerate the nuclear translocation of p65 in A375 cells (Figure 3E). Next, we transfected A375 cells with Casp8 plasmids in the presence of several inhibitors, Nec‐1s (RIP1), GSK872 (RIP3), necrosulfonamide (MLKL), and BAY‐11‐7082 (NF‐kappaB) for 24 hours and detected the expression of A20. We found that only BAY‐11‐7082 abolished Casp8‐induced A20 expression (Figure 3F, lane 6). To further dissect these findings, siRNAs were transfected into cells to knock down the transcription factor p65 in the presence of Casp8, and the Western blot results indicated that decreased p65 impeded A20 expression (Figure 3G, lane 1 and lane 3) and abrogated Casp8‐induced A20 upregulation (Figure 3G, lanes 1‐4; Figure S3D). Intriguingly, we found that knocking down RIP1, which can be cleaved by active Casp8,^35^ significantly increased A20 expression (Figure 3G, lane 1 and lane 5). Western blot analysis also showed that Casp8 decreased RIP1 expression in A375 cells (Figure 3H). All these results indicated that the activation of NF‐kappaB and decrease in RIP1 both contributed to Casp8‐increased A20 expression (Figure 3I).

**FIGURE 4:**
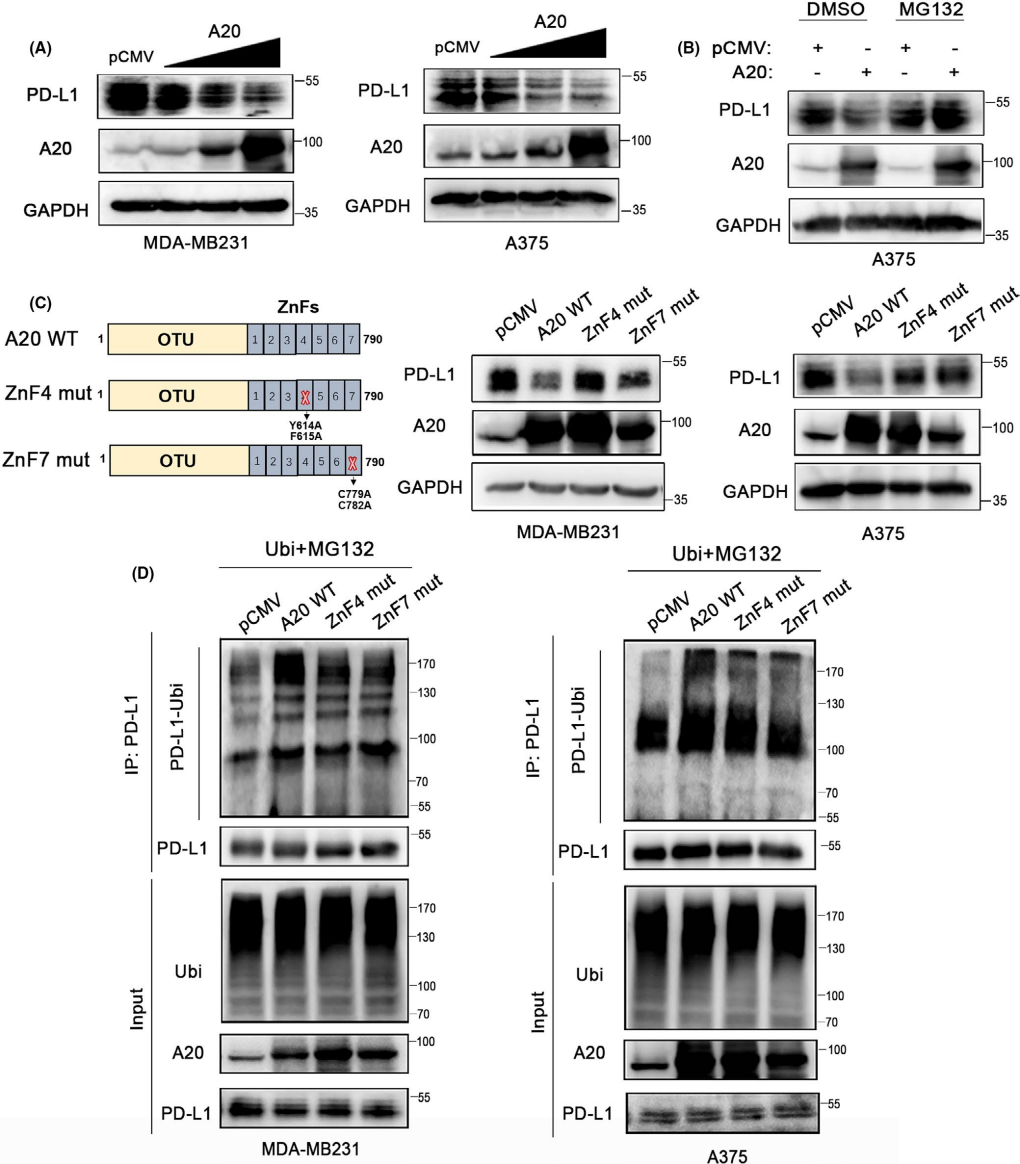
A20 facilitates the ubiquitination of the PD‐L1 protein. A, MDA‐MB231 and A375 cells were transfected with A20 plasmids with different concentration gradients (0.5 µg, 1 µg, 1.5 µg, and 2 µg) for 48 h, and PD‐L1 expression was measured by Western blot analysis. B, Western blot analysis of PD‐L1 expression in A375 cells transfected with A20 plasmids in the presence of MG132 (3 µmol/L) for 48 h. C, A20‐WT, the ZnF4 mutant, and the ZnF7 mutant were transfected into MDA‐MB231 and A375 cells, respectively. Western blot analysis was performed to measure the expression of PD‐L1 48 h after transfection. D, Analysis of ubiquitin PD‐L1 in MDA‐MB231 and A375 cells, which were transfected with A20‐WT, the ZnF4 mutant, or the ZnF7 mutant for 24 h, followed by treatment with MG132 (3 µmol/L) for 24 h

Subsequently, we wondered whether A20 interacts with PD‐L1 and leads to its ubiquitination. A20 plasmids of different dose gradients were transfected into MDA‐MB231, A375, and 293T cells for 24 hours. The expression of PD‐L1 was detected by Western blot. The results suggested that A20 promoted PD‐L1 protein degradation in a dose‐dependent manner (Figure 4A), and this degradation was attenuated in the presence of MG132 (Figure 4B). According to previous studies, the C‐terminal region of the A20 protein contains seven zinc fingers (ZnFs), which are required for the assembly of polyubiquitin chains. ZnF3‐4 regions are the minimal portion of the protein that was required for A20 ubiquitin activity, with ZnF4 being essential.^36^ In addition to the ZnF4 region, the ZnF7 region of A20 has also been implicated in regulating TNFR1 signaling and inflammatory disease through its ubiquitin activity.^37^, ^38^ To identify which regions of A20 contribute to PD‐L1 ubiquitination, we performed site‐directed mutagenesis assays of the ZnF4 or ZnF7 region in A20 to obtain the ZnF4 mutant and the ZnF7 mutant. Next, wild‐type A20 and mutant A20 were transfected into cells, and changes in the PD‐L1 protein were measured. The results showed that the ZnF4 mutant and the ZnF7 mutant cannot reduce PD‐L1 expression as wild‐type A20 did (Figure 4C). Co‐immunoprecipitation (Co‐IP) assays also showed that the ZnF4 mutant and the ZnF7 mutant failed to generate significant amounts of ubiquitin chains compared with wild‐type A20 (Figure 4D). In conclusion, these results indicated that the ubiquitin‐binding domains ZnF4 and ZnF7 of A20 both played a crucial role in A20‐mediated PD‐L1 ubiquitination.

### 3.4 A20 is required for Casp8‐induced PD‐L1 degradation

**FIGURE 5:**
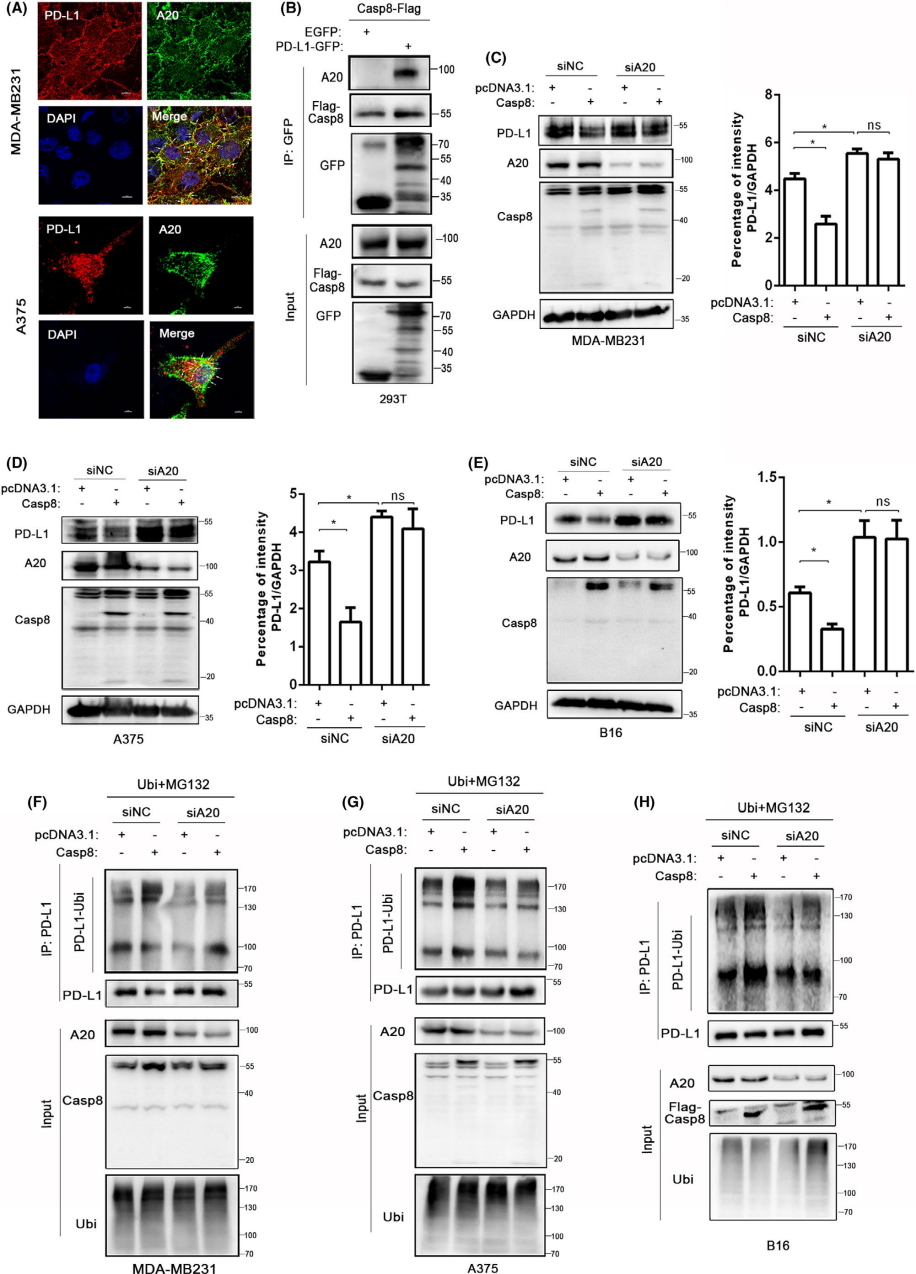
A20 is required for Casp8‐induced PD‐L1 degradation. A, Confocal microscopy image showing the protein expression of PD‐L1 and A20 in MDA‐MB231 and A375 cells. Scale bar: 10 µm for the MDA‐MB231 cells and 5 µm for the A375 cells. B, Immunoprecipitation assay analysis of the interaction of PD‐L1 and A20 or Casp8. The cells were cotransfected with PD‐L1‐GFP and Casp8‐Flag plasmids, and exogenous Casp8 and endogenous A20 were immunoprecipitated with anti‐GFP antibody. C‐E, Western blot analysis of the expression of PD‐L1 in MDA‐MB231 (C), A375 (D), and B16 cells (E) after cotransfection with siA20 and Casp8 plasmids for 48 h. ImageJ was used to analyze the gray of lanes. Histogram indicated the intensity of PD‐L1/GAPDH. *P* <.05 (*), unpaired two‐tailed *t*‐test. F‐H, Ubiquitination assays of PD‐L1 in MDA‐MB231 (F), A375 (G), and B16 (H) cells transfected with siA20 and Casp8 plasmids. Ubiquitin PD‐L1 was immunoprecipitated with an anti‐PD‐L1 antibody and subjected to Western blotting with an anti‐ubiquitin antibody

Our previous results showed that Casp8 colocalized with the PD‐L1 protein (Figure 1H). To determine whether there is a correlation between Casp8, A20, and PD‐L1, we investigated whether PD‐L1 colocalizes with A20. Colocalization of PD‐L1 and A20 was confirmed in MDA‐MB231 and A375 cells by immunofluorescence double staining (Figure 5A). To verify their interaction, we performed a co‐IP assay with 293T cells, and the results suggested that both Casp8 and A20 can be pulled down by anti‐GFP‐PD‐L1 beads (Figure 5B). Next, we examined the regulatory roles of A20 in Casp8‐induced PD‐L1 ubiquitin degradation. We decreased A20 protein expression through siRNAs. MDA‐MB231, A375, B16, and His‐PD‐L1–expressing 293T cells were cotransfected with Casp8 plasmids and siA20 for 48 hours. Western blot results showed that A20 downregulation rescued Casp8‐induced PD‐L1 degradation (Figure 5C‐E, Figure S3E). Indeed, the co‐IP experiments performed to identify PD‐L1 interactors suggested that Casp8 facilitates the recruitment of ubiquitin chains to PD‐L1, while knocking down A20 restores PD‐L1 ubiquitination (Figure 5F‐H). All these results suggested that Casp8 induced PD‐L1 ubiquitination indirectly but acted in an A20‐dependent manner.

### 3.5 Knocking down Casp8 suppresses tumor immunogenicity by upregulating PD‐L1

**FIGURE 6:**
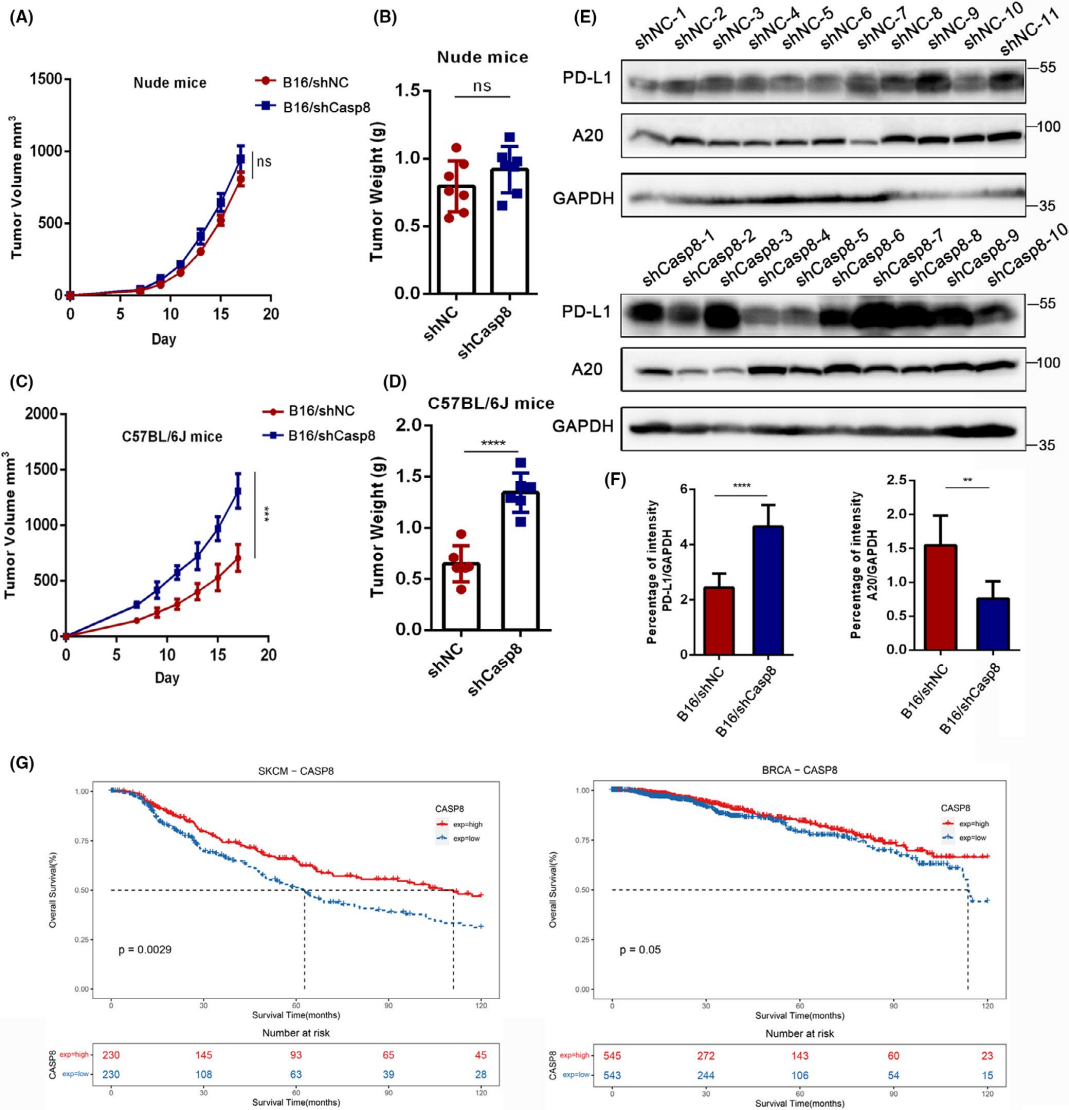
Knocking down Casp8 suppresses tumor immunogenicity by upregulating PD‐L1. A‐D, Nude mice (A, B) (n = 7 per group) were injected subcutaneously with B16/shNC or B16/shCasp8 cells (1.5*10^5^); C57BL/6J mice (C, D) (n = 6 per group) were injected subcutaneously with B16/shNC or B16/shCasp8 cells (2 * 10^5^). Tumor volume (A, C) and tumor weight (B, D) were measured at the indicated times. Differences that were not significant are denoted with by ns, *P*‐value <.001 (***); unpaired two‐tailed *t*‐test. E, Western blot analysis of PD‐L1 and A20 expression in tumor tissues removed from the mice mentioned in A‐D. The samples were obtained from two independent experiments. The two groups were exposed to chemiluminescence for the same length of time. F, PD‐L1 and A20 expression in the tumor tissues removed from mice was quantified by densitometry. *P*‐value <.01 (**) and <.001 (***); unpaired two‐tailed *t*‐test. G, Kaplan‐Meier curves from the survival analysis based on Casp8 expression levels in skin melanoma patients and breast cancer patients. The data were downloaded from TCGA and analyzed by R studio software

To explore the roles of Casp8 in tumor progression with or without a complete immune system, we screened murine B16 cells after Casp8 knockdown and injected them s.c. into mice. In nude mice, we observed that there was no significant difference between tumor growth in the Casp8‐knockdown (B16/shCasp8) group and that of the control (B16/shNC) group (Figure 6A); similarly, no difference in tumor weight was found (Figure 6B). In immunocompetent C57BL/6J mice, we found that knocking down Casp8 significantly promoted tumor growth and led to increased tumor volume (Figure 6C and D). We analyzed the function of tumor‐infiltrating CD8^+^ T cells in C57BL/6J mice with injection of B16/shNC or B16/shCasp8 cells. The results showed that the CD8^+^ T cells obtained from the Casp8‐knockdown group produced a lower frequency of the cytokine IFN‐γ than those from the control group (Figure S4A). Western blotting was performed to measure the PD‐L1 and A20 expression in tumor tissues resected from each mouse. As expected, PD‐L1 expression levels were significantly upregulated in tumors derived from B16/shCasp8 cells, while A20 expression was decreased (Figure 6E and F). We also screened murine 4T1 cells and performed the assay in BALB/c mice. The results were consistent with the above conclusion (Figure S4B). Next, to determine the association between Casp8 expression and the prognosis of cancer patients, we performed Kaplan‐Meier survival analyses and found that low Casp8 expression was correlated with worse overall survival of patients with skin melanoma, breast cancer, rectal adenocarcinoma, and ovarian carcinoma based on the data obtained from TCGA database (Figure 6G, Figure S4C). These findings indicated that low Casp8 expression promoted cancer progression in an immune system–dependent manner.

### 3.6 Knocking out Casp8 results in dysfunctional NK cells and facilitates therapeutic sensitivity to mAb therapy

**FIGURE 7:**
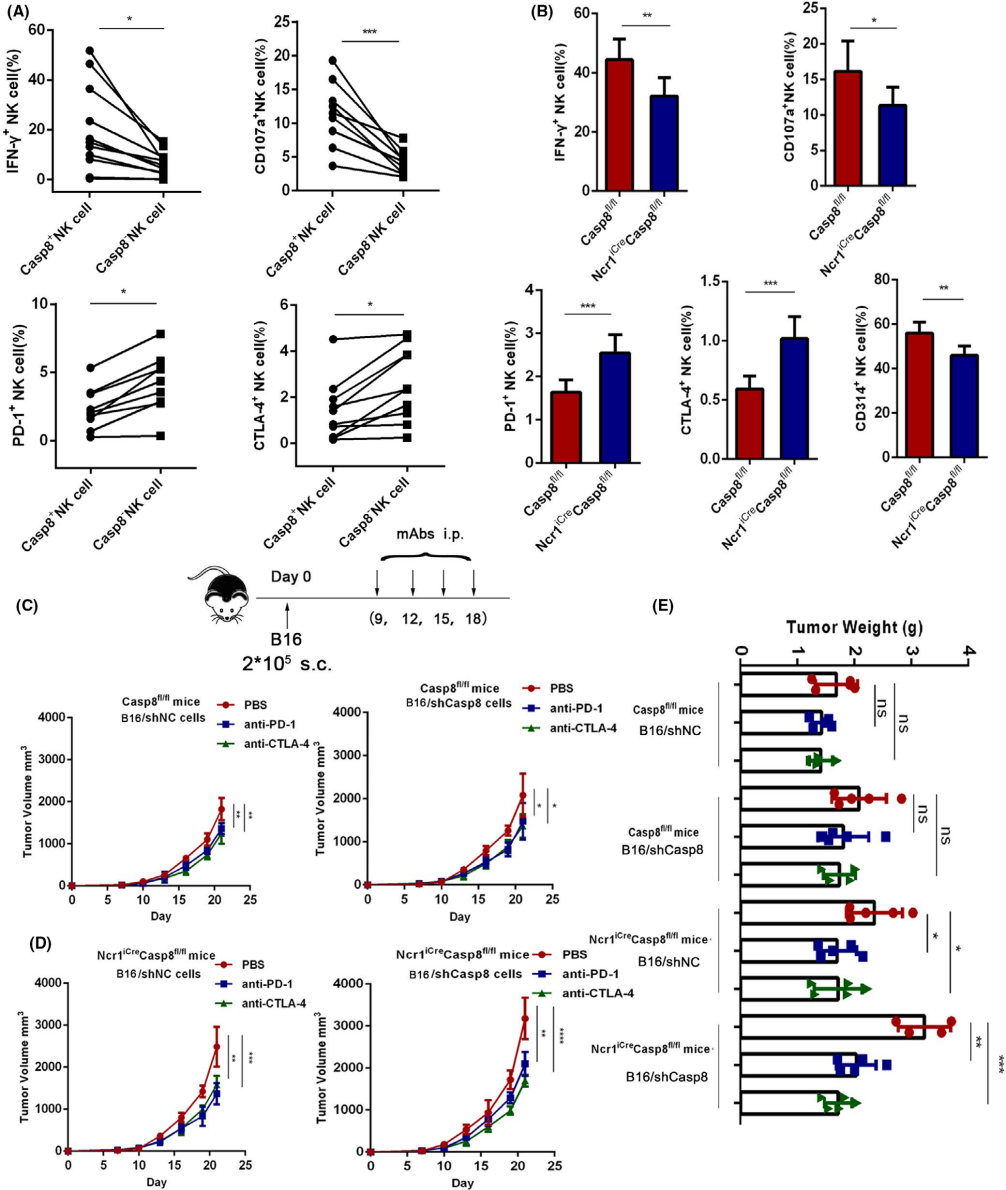
Knocking out Casp8 results in NK cell dysfunction and facilitates therapeutic sensitivity to monoclonal antibody therapy. A, Flow cytometry analysis of the frequency of cells expressing IFN‐γ, CD107a, PD‐1, and CTLA‐4 in Casp8^+^ NK cells and Casp8^‐^ NK cells among the PBMCs from cancer patients, *P‐*value <.05 (*), <.001 (***). B, Frequency of cells expressing IFN‐γ, CD107a, PD‐1, CTLA‐4, and CD314 among splenic NK cells from Casp8^fl/fl^ mice (n = 9) and Ncr1^iCre/+^Casp8^fl/fl^ mice (n = 6), *P*‐value <.05 (*), <.01 (**), and <.001 (***). C, D, The tumor volume of Casp8^fl/fl^ mice (C) and Ncr1^iCre/+^Casp8^fl/fl^ mice (D) were intraperitoneally (i.p.) injected with PBS, anti‐PD‐1, and anti‐CTLA‐4 at the 9th, 12th, 15th, and 18th day after subcutaneous injection with 2 * 10^5^ B16/shNC or B16/shCasp8 melanoma cells at day 0. E, Tumor weights of the mice treated as indicated in C and D, *P‐*values <.05 (*), <.01 (**), and <.001 (***); differences that were not significant are denoted by ns

Accumulating evidence indicates that Casp8 plays an essential role in the activation of T cells and B cells, and T cells with loss of Casp8 fail to respond to antigenic stimulation, resulting in a deficient immune response.^23^, ^39^, ^40^ However, the functions of Casp8 in NK cells remain unclear. To validate the functions of Casp8 in the NK cells of cancer patients, we used flow cytometry to analyze PBMCs of patients with advanced cancer. Comparing the phenotype of Casp8^+^ NK cell subsets with that of Casp8^‐^ NK cell subsets, we found that a higher frequency of Casp8^+^ NK cells than Casp8^−^ NK cells expressed the cytokine IFN‐γ and the lysosome marker CD107a, which suggested that lower expression of Casp8 in NK cells reduced effector function and anticancer potency. Moreover, we also found that Casp8^−^ NK cells had higher expression of PD‐1 and CTLA‐4 on the surface, which signified that anti‐PD‐1 and anti‐CTLA‐4 therapies might attenuate the dysfunction of these NK cells (Figure 7A, Figure S5A). To validate our results, immunofluorescence double staining of pathological tissues from patients with colorectal cancer showed that the infiltration of NK cells in tumor tissues was greater than that in normal tissues. Additionally, tumor‐infiltrating NK cells expressed lower Casp8 levels than normal tissues (Figure S5B). Furthermore, we bred Casp8^fl/fl^ mice with Ncr1^iCre^ mice to obtain offspring with abrogated Casp8 expression specifically and exclusively in NK cells (Ncr1^iCre/+^Casp8^fl/fl^ mice). The genotyping results are shown in Figure S5C. Using flow cytometry, we analyzed the function of NK cells derived from the spleen of Casp8^fl/fl^ mice (control) and Ncr1^iCre/+^Casp8^fl/fl^ mice, and the results suggested that Casp8^fl/fl^ mice produced the cytokine IFN‐γ and the lysosome marker CD107a at a higher frequency than Ncr1^iCre/+^Casp8^fl/fl^ mice, while the change in the inhibitory receptor NKG2A was not significant (Figure 7B, Figure S6A and B). Consistent with human samples, Ncr1^iCre/+^Casp8^fl/fl^ mice also had higher expression of PD‐1 and CTLA‐4 (Figure 7B). All results indicated that deficiency of Casp8 in NK cells lead to functional defects in NK cells, while anti‐PD‐1 or anti‐CTLA‐4 treatments might alleviate this dysfunction. To verify this conclusion, we injected B16/shNC cells or B16/shCasp8 cells s.c. into Casp8^fl/fl^ mice or Ncr1^iCre/+^Casp8^fl/fl^ mice as design. After 9 days, the tumor‐bearing mice were treated with anti‐PD‐1 or anti‐CTLA‐4 antibodies. the tumor volume of the mice was monitored. Consistent with the results described above, B16/shCasp8 cell–derived tumor growth was greater than the growth of tumors derived from B16/shNC cells in both the Casp8^fl/fl^ mice and Ncr1^iCre/+^Casp8^fl/fl^ mice; but compared with the Ncr1^iCre/+^Casp8^fl/fl^ mice, the tumor volume and weight of the Casp8^fl/fl^ mice were smaller. As expected, after treatment with anti‐PD‐1 or anti‐CTLA‐4, the degree of tumor reduction in the Ncr1^iCre/+^Casp8^fl/fl^ mice was more significant than that in the Casp8^fl/fl^ mice (Figure 7C‐E). The expression of Granzyme B in the tumor tissues resected from each mouse was measured by Western blot to evaluate the functions of immune cells (Figure S6C and D). All these results showed that Casp8 played a critical role in tumor progression. Downregulation of Casp8 in both the cancer cells and/or NK cells may lead to severe immune tolerance because of the upregulation of PD‐L1 and/or PD‐1 and CTLA‐4. These negative effects were alleviated by anti‐PD‐1 and anti‐CTLA‐4 therapies, which indicated that knocking down Casp8 might enhance the sensitivity of these cells to mAb therapies. In summary, our data indicated that cancer patients with low Casp8 expression might benefit the most from anti‐PD‐1 or anti‐CTLA‐4 immunotherapy.

## 4 DISCUSSION

The expression of PD‐L1 has been suggested to be associated with the efficacy of anti‐PD‐L1/PD‐1 therapy, and elevated PD‐L1 expression in tumor cells causes the exhaustion of CTLs.^41^ Recently, many studies have suggested that PTMs of PD‐L1 play critical roles in PD‐L1–mediated immunosuppression, and multiple small‐molecule inhibitors, natural food compounds, and mAbs have been identified with anticancer potential because they target the PTM marks of PD‐L1.^42^ In our study, we found a novel mechanism of PD‐L1 PTMs induced by the Casp8/A20 axis, which may provide a new therapeutic strategy for mAb treatments. A20 is a bifunctional ubiquitin‐editing enzyme acting as a deubiquitinase or ubiquitin ligase that can terminate cytokine‐induced NF‐kappaB signaling pathways. Previous studies demonstrated that the polyubiquitination of Casp8 induced by Apo2L/TRAIL can be attenuated by A20.^32^ A20 also stably interacts with Casp8 in human T cell leukemia virus type I (HTLV‐I)–infected cells, thereby protecting them from apoptosis.^43^ We determined that Casp8 increased A20 expression by activating NF‐kappaB and cleaving RIP1. Our results do not necessarily contradict the findings showing direct regulation of PD‐L1 expression by NF‐kappaB. In our research, Casp8‐induced A20 expression did not rely only on NF‐kappaB, and decreasing RIP1 also increased A20 protein expression, thus providing negative feedback control of NF‐kappaB signaling. The PTMs of the PD‐L1 protein induced by A20 played a more dominant role than NF‐kappaB in our models.

Previous studies have indicated that cancers with low MHC expression are responsive to checkpoint blockade treatment, suggesting that other types of immune cells may also be involved in checkpoint‐based antitumor immunotherapy.^44^ Casp8 plays a critical role in activating T cells and B cells,^22^, ^23^, ^39^, ^40^ while the functions of Casp8 in NK cells remain unclear. In our research, we found that the deletion of Casp8 in NK cells resulted in the dysfunction of the NK cells, but it also increased the expression of PD‐1 and CTLA‐4 on NK cytomembranes, which has been reported to be associated with poor prognosis in various cancers.^45^, ^46^, ^47^, ^48^ After treatment with anti‐PD‐1/PD‐L1 antibodies, PD‐1^+^ NK cells have a higher frequency of CD107a and IFN‐γ expression than PD‐1^‐^ NK cells,^48^ suggesting that NK cell dysfunction may be alleviated by anti‐PD‐1 or anti‐CTLA‐4. The results from mouse immunotherapy assays validated our hypothesis.

Several studies have suggested that Casp8 mutations in cancers lead to differences in Casp8 protein expression, with most mutations resulting in a decrease in Casp8 protein expression.^24^, ^27^, ^28^ We found that mice with low Casp8 expression were more sensitive to mAb therapy. Though the tumor volume and weight of the Ncr1^iCre/+^Casp8^fl/fl^ mice were still slightly larger than those of the Casp8^fl/fl^ mice, the degree of tumor reduction in the Ncr1^iCre/+^Casp8^fl/fl^ mice was more significant than that in the Casp8^fl/fl^ mice. However, multiple types of Casp8 mutations may lead to differences in the Casp8 expression level,^24^ and further research is required to confirm the correlation between PD‐L1 and different types of Casp8 mutants. The potential roles of CD8+ T cells might also affect the curative effect of mAbs, but we found no significant difference in PD‐1 and CTLA‐4 expression in the CD8+ T cells of Casp8^fl/fl^ mice and Ncr1^iCre/+^Casp8^fl/fl^ mice. Our data suggest a novel therapeutic indicator for evaluating the sensitivity of anti‐PD‐1 or anti‐CTLA‐4 treatments in patients with cancer, and further studies should be applied to validate these results in clinical trials.

## CONFLICT OF INTEREST

The authors declare no competing financial interests.

## Supporting information

Appendix S1
