# Supplementary material for: Casp8 acts through A20 to inhibit PD‐L1 expression: The mechanism and its implication in immunotherapy
Source: Cancer Sci. 2021 May 20;112(7):2664–78. doi: 10.1111/cas.14932 (PMC8253292; doi:10.1111/cas.14932)
Supplement: Supplementary file 1 — Appendix S1 [file CAS-112-2664-s001.docx]

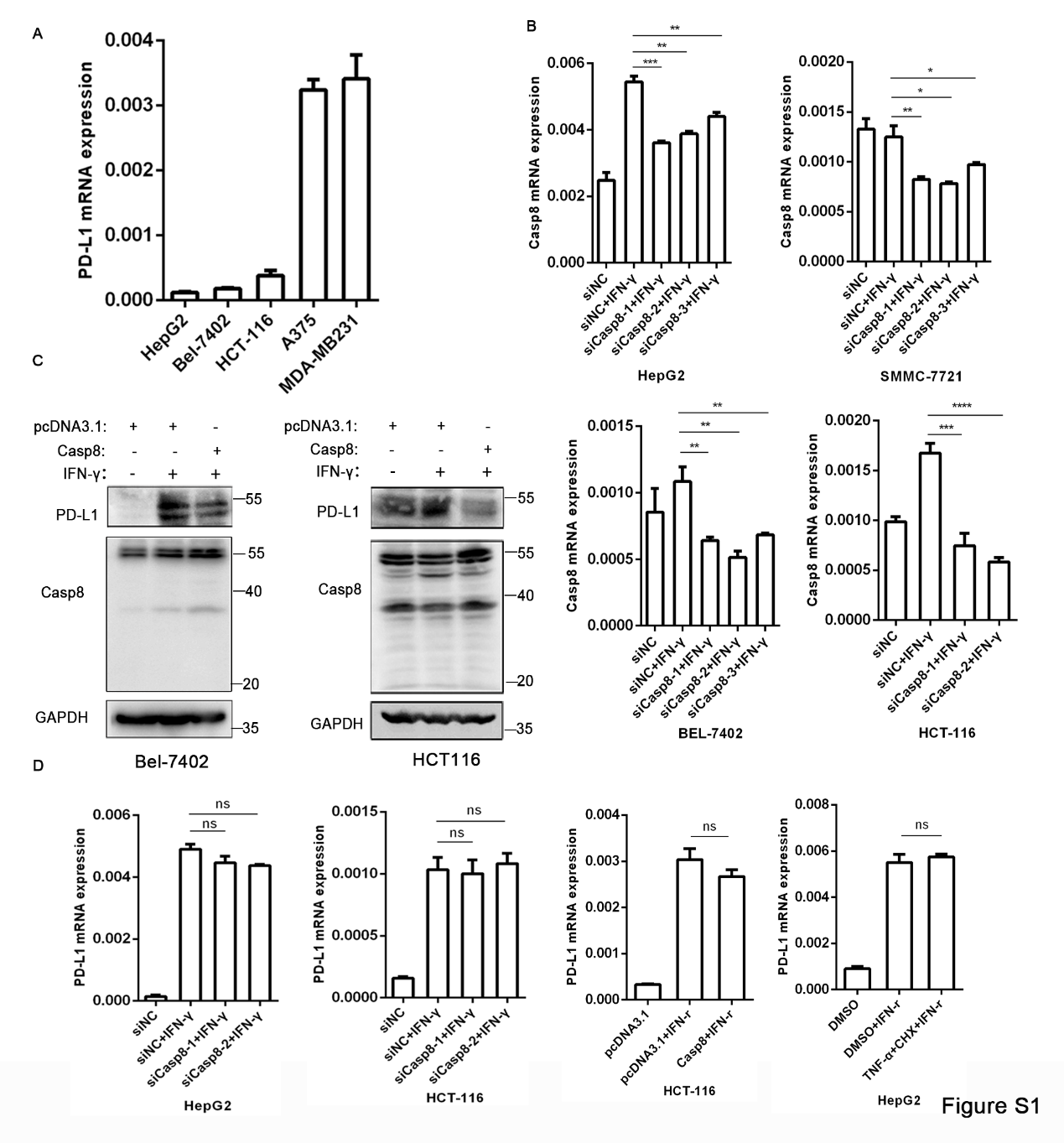


**Figure S1: Related to Figure 1, Casp8 downregulates PD-L1 protein expression**

Fig S1A: RT-PCR analysis of the PD-L1 expression in HepG2, Bel-7402, HCT-116, A375, MDA-MB231 cells.

S1B: RT-PCR analysis of the efficacy of Casp8 siRNAs in HepG2, SMMC-7721, Bel-7402, HCT-116 cells.

S1C: Western blot analysis IFN-γ induced PD-L1 expression in Bel-7402 and HCT-116 cells transfected with Casp8 plasmids.

S1D: The cells were transfected with Casp8 plasmids or siRNAs or stimulated by TNF-α and CHX in the present of IFN-γ (50 ng/ml), RT-PCR analyzed PD-L1 expression at mRNA level.


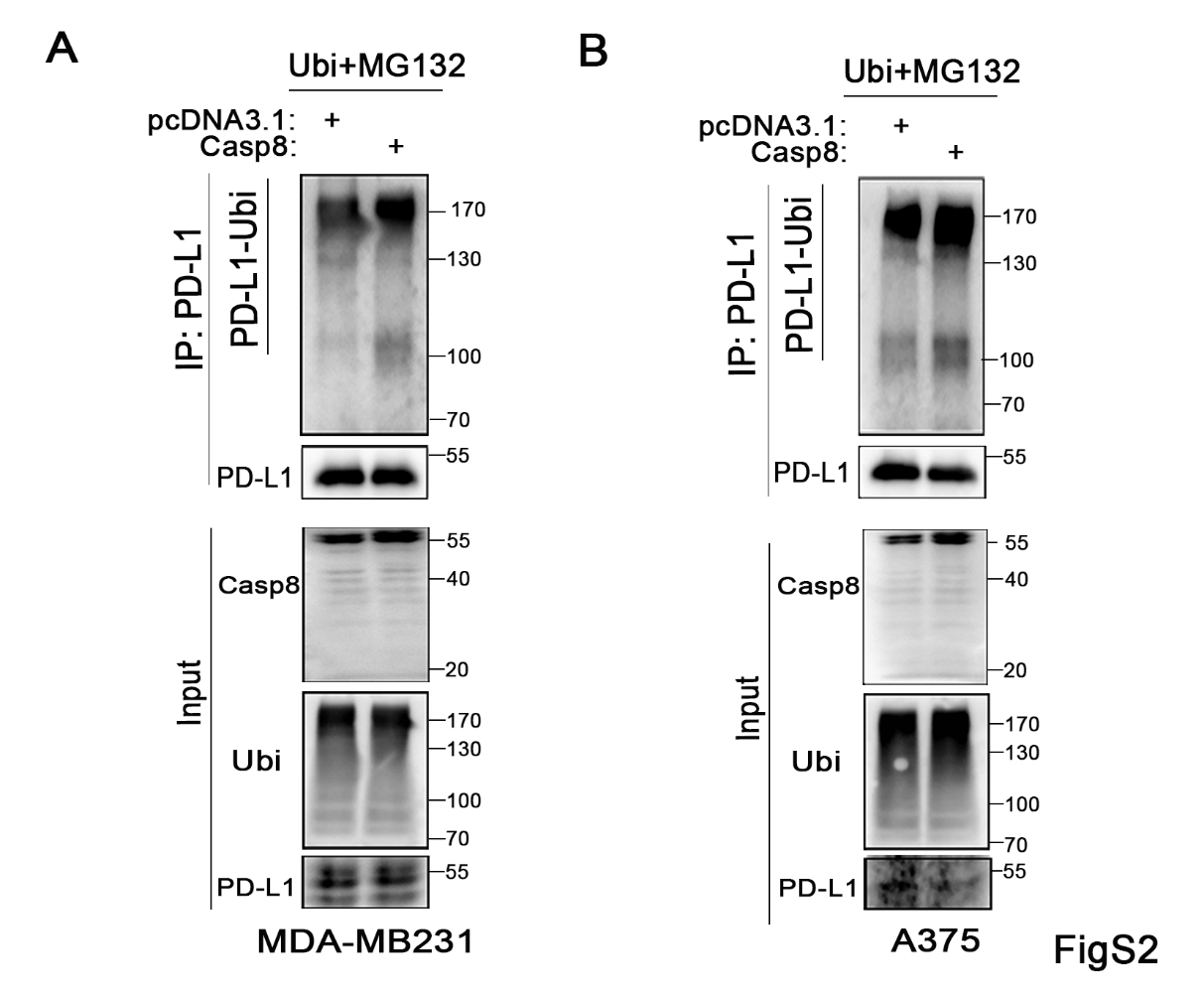


**Figure S2: Related to Figure 2, Activated Casp8 induces ubiquitination of the PD-L1 protein**

Fig S2A-B: Ubiquitination assay of PD-L1 in MDA-MB231 (A) and A375 (B) cells. Cells were transfected with various plasmid as design and treated with DMSO or MG132 (3µM) for 24h. Ubiquitinated PD-L1 was pulled-down by anti-6xHis tag antibody and subjected to western blot analysis by anti-ubiquitin antibody.


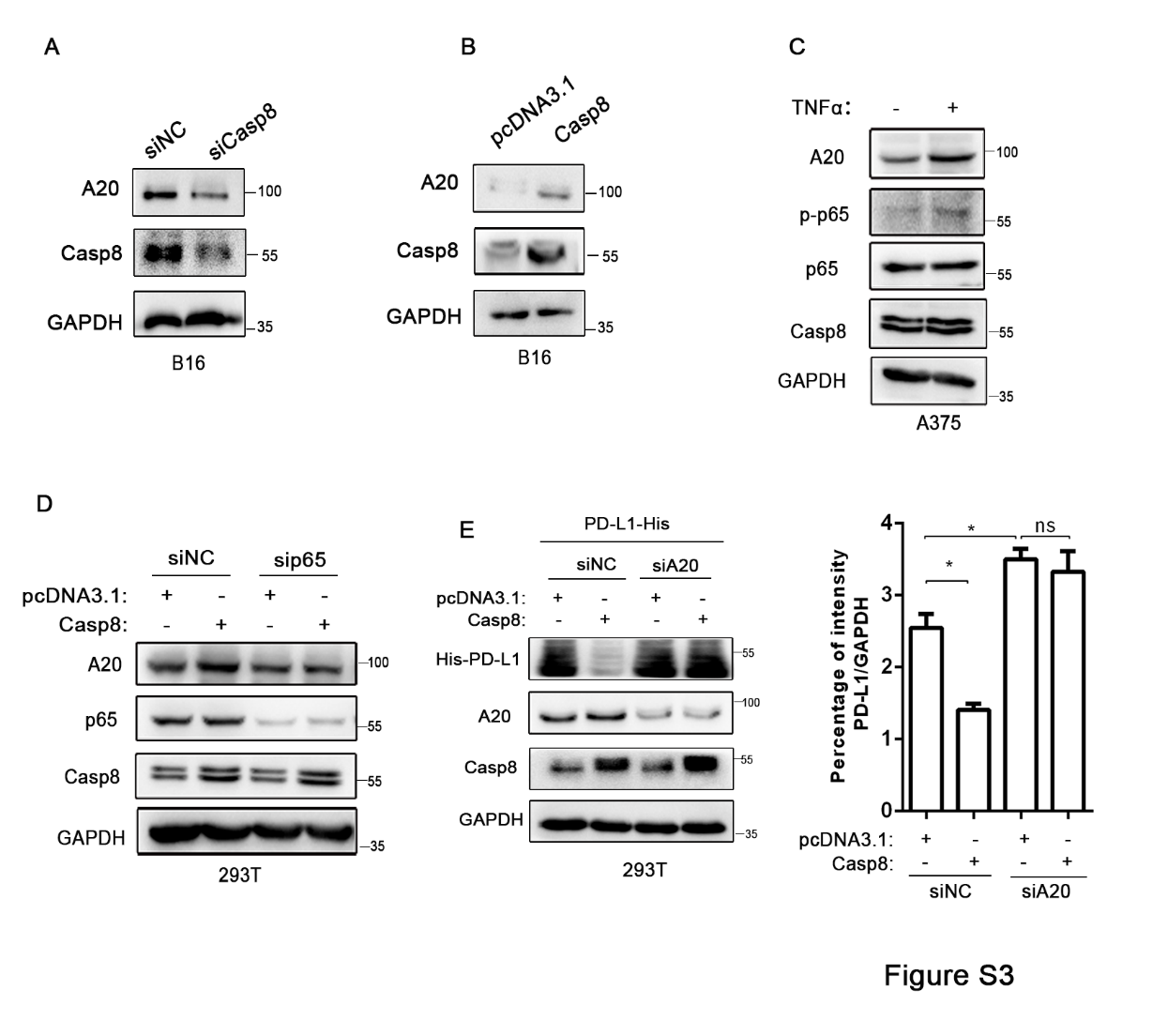


**Figure S3: Related to Figure 3 and Figure 5, A20 is required for Casp8-induced PD-L1 degradation**

Fig S3A-S3B: B16 cells were transfected with siCasp8 (A) or Casp8 plasmids (B) for 48 h or 24 h, western blot were used to detected the A20 expression.

S3C: A375 cells were treated with TNF-α (10ng/ml) for 24h to active NF-kappaB pathway, Western blot was used to analyze A20, p-p65, Casp8 expression.

S3D: 293T cells were transfected with sip65 for 24h, followed by transfection of pcDNA3.1 or Casp8 for 24h, western blot were performed to detected the A20 expression.

S3E: Western blot analysis of the expression of His-PD-L1 expression in His-PD-L1 expressing 293T cells after co-transfection with siA20 and Casp8 plasmids for 48 h. ImageJ was used to analyze the gray of lanes, the histogram indicated intensity of PD-L1/GAPDH. p <0.05 (*), unpaired two‐tailed t‐test.


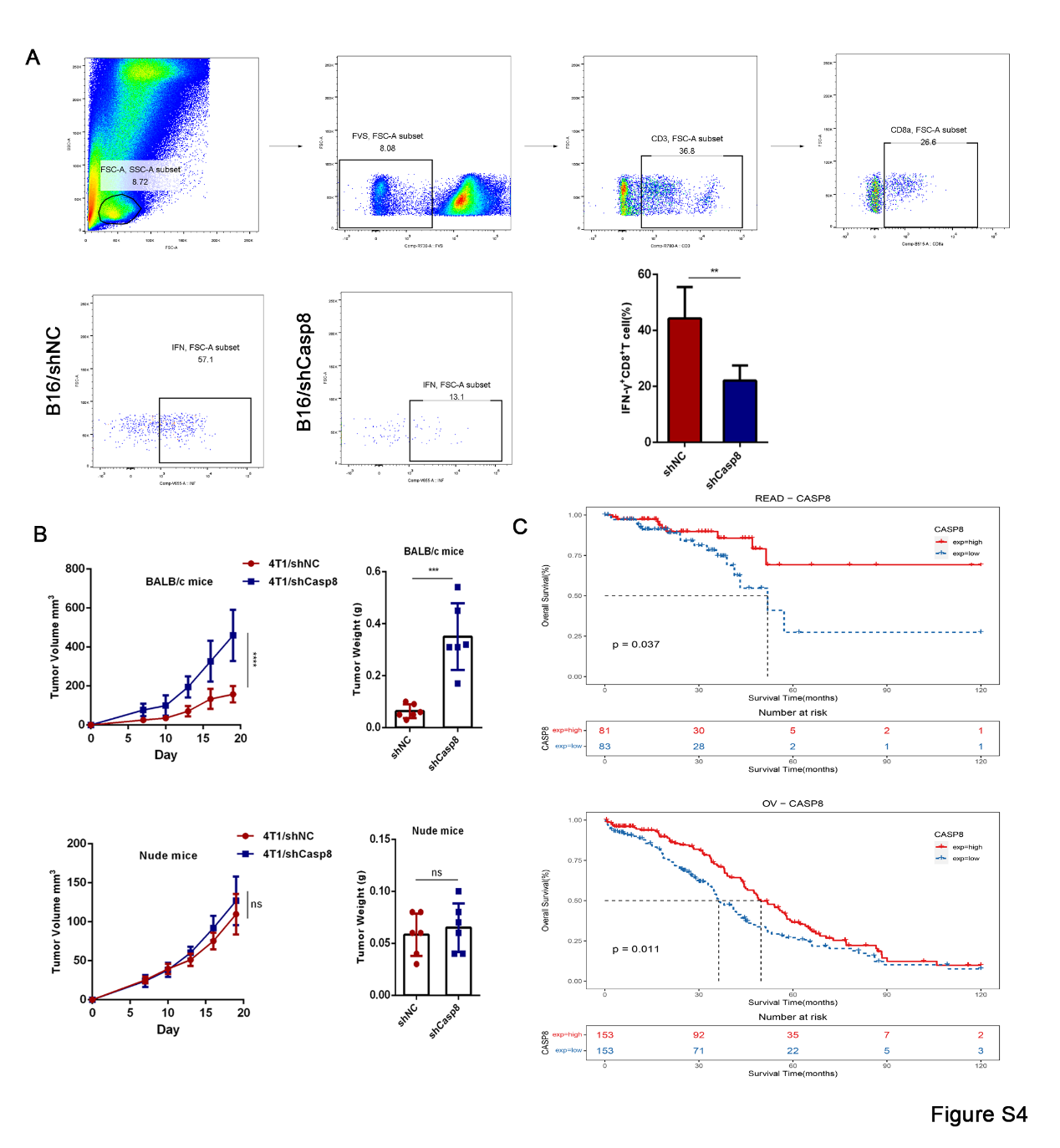


**Figure S4: Related to Figure 6, Knocking down Casp8 suppresses tumor immunogenicity by upregulating PD-L1**

Fig S4A: Flow cytometry analysis of the IFN-γ expression of tumor infiltrating CD8^+^ T cells obtain from mice (n=5 per group) injected with B16/shNC or B16/shCasp8 cells.

S4B: BALB/c (n=6 per group) or nude mice (n=6 per group) were injected subcutaneously with 4T1/shNC or B16/shCasp8 cells, tumor volume and tumor weight were measured at the indicated times. Differences that were not significant are denoted with by ns, *p* value <0.001 (***); unpaired two‐tailed t‐test.

S4C: Kaplan-Meier curves from the survival analysis based on Casp8 expression levels in rectal adenocarcinoma patients and ovarian carcinoma patients. The data were downloaded from the TCGA and analyzed by R studio software.


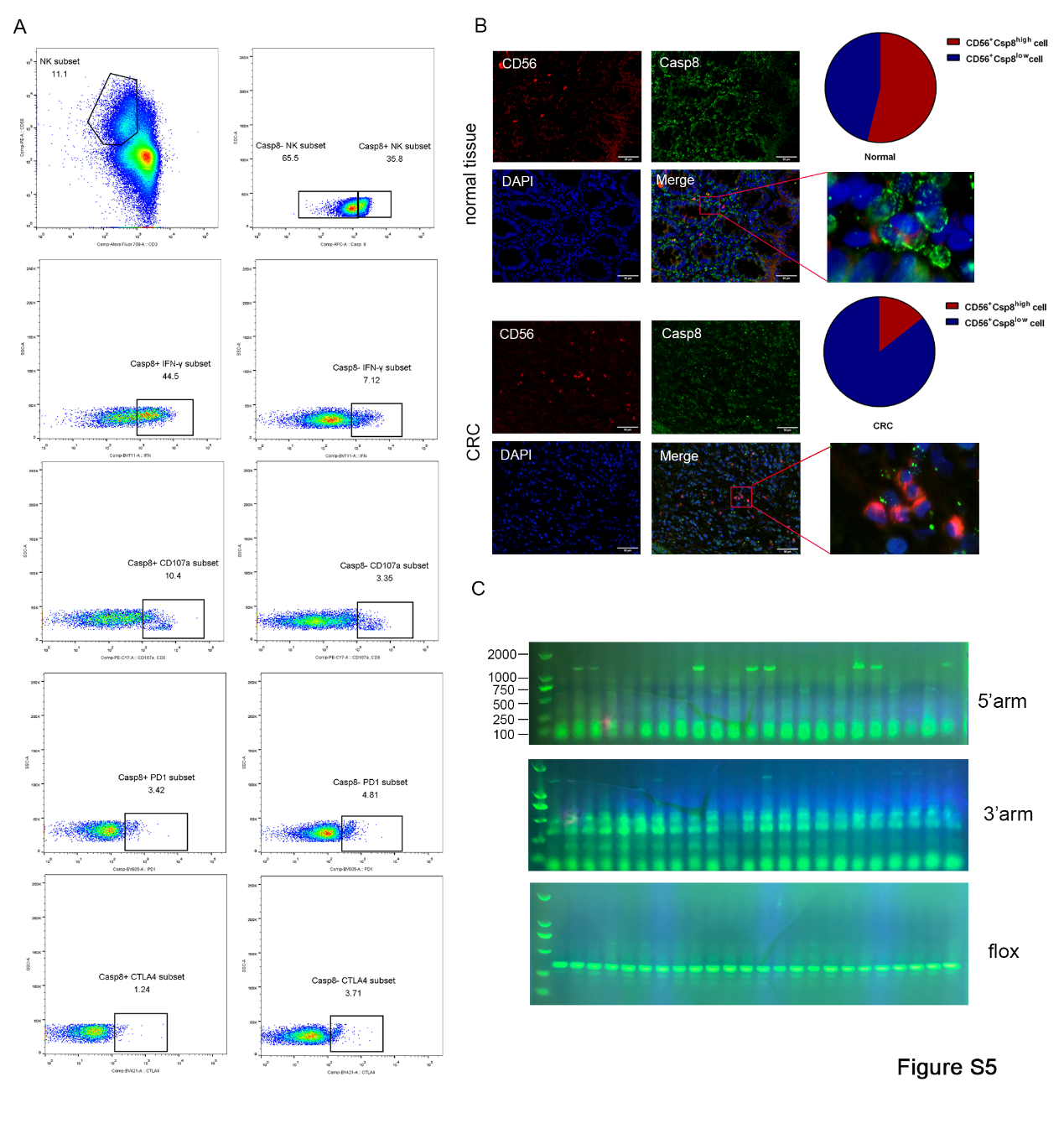


**Figure S5: Related to Figure 7, Knocking out Casp8 results in human NK cell dysfunction**

Figure S5A: Flow cytometry analyzed frequency of cells expressing IFN-γ, CD107a,PD-1,CTLA-4 among Casp8^+^NK cells and Casp8^-^ NK cells from patients PBMC.

S5B: Double immunofluorescence staining of tumor-infiltrating NK (CD56^+^) and Casp8 in human normal tissues and CRC tissues(n=10 per group) . (CD56^+^Casp8^high^: NK cells with high Casp8 expression; CD56^+^Casp8^low^: NK cells with low Casp8 expression.

S5C: Genotyping of Casp8^fl/fl^ mice and NcrI^iCre^ Casp8^fl/fl^ mice.


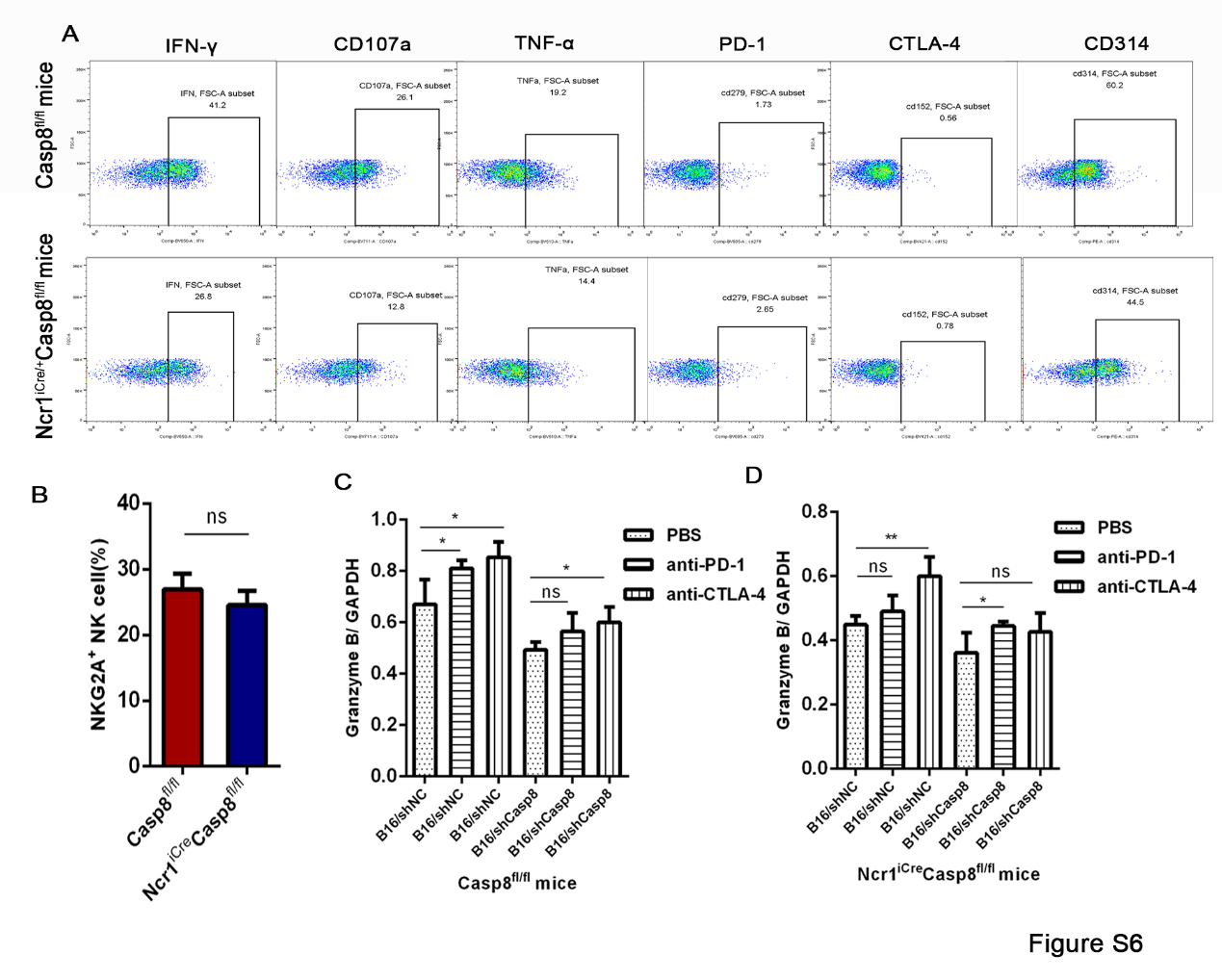


**Figure S6: Related to Figure 7, Knocking out Casp8 in NK cell facilitates therapeutic sensitivity to monoclonal antibody therapy.**

Fig S6A: Frequency of cells expressing IFN-γ, CD107a, TNF-α, PD-1(CD279), CTLA-4(CD152) and CD314 among splenic NK cells from Casp8^fl/fl^ mice (n=9) and NcrI^iCre^ Casp8^fl/fl^mice (n=6).

S6B: Frequency of cells expressing NKG2A among splenic NK cells from Casp8^fl/fl^ mice (n=9) and NcrI^iCre^ Casp8^fl/fl^mice (n=6).

S6C: The survival of mice with treatment of PBS, anti-PD-1, anti-CTLA-4 mentioned in Figure7C.

S6D: Western blot analysis of the expression of Granzyme B in tumor tissues removed from mice showed in Figure7C.

**Supplement Table 1: Antibodies involved in manuscript**

| **Brand name** | **Antibody name** | **Cat No.** | **Application** |
| --- | --- | --- | --- |
| Cell Signaling Technology | anti-PD-L1 antibody | #13684 | WB, IHC |
| Abcam | anti-PD-L1 antibody | ab213524 | IF |
| Proteintech Group | anti-PD-L1 antibody | 17952-1-AP | Co-IP |
| Cell Signaling Technology | anti-Casp8 antibody | #9746 | WB |
| Proteintech Group | anti-Casp8 antibody | 66093-1-Ig | IF, IHC |
| Biorbyt | anti-Casp8 antibody | orb10241 | FACS |
| HUABIO | anti-A20 antibody | ET1611-40 | WB, IHC |
| Proteintech Group | anti-A20 antibody | 66695-1-Ig | IF |
| Cell Signaling Technology | anti-A20 antibody | #5630 | WB, Co-IP |
| Abcam | anti-ubiquitin antibody | ab134953 | WB |
| BBI | anti-6xHis tag | D191001 | WB |
| Proteintech Group | anti-Flag-antibody | 66008-3-Ig | WB |
| HUABIO | anti-NF-kappaB p65 | ET1603-12 | WB |
| HUABIO | anti-PhosphoNF-kB-p65(S529) | ET1604-27 | WB |
| GeneTex | anti-p84 antibody | GTX70220 | WB |
| ZSGB-BIO | anti-GAPDH antibody | TA-08 | WB |
| Proteintech Group | anti-β actin antibody | 66009-1-Ig | WB |
| Abcam | anti-RIP1 antibody | ab125072 | WB |
| Abcam | anti-Granzyme B | ab255598 | WB |
| BD | Alexa Fluor 700 conjugated anti-human CD3 antibody | 557917 | FACS |
| BD | PE conjugated anti-human CD56 antibody | 561903 | FACS |
| BD | BV421 conjugated anti-human CD152 antibody | 562743 | FACS |
| BD | BV605 conjugated anti-human PD-1 antibody | 563245 | FACS |
| BD | PE-CY7 conjugated anti-human CD107a antibody | 561348 | FACS |
| BD | BV711 conjugated anti-human IFN-γ antibody | 564039 | FACS |
| Biolegend | PE conjugated anti-human PD-L1 antibody | 329705 | FACS |
| Absin | APC conjugated anti-rabbit IgG antibody | abs20032 | FACS |
| BD | APC-Cy7 conjugated anti-mouse CD3 antibody | 560590 | FACS |
| BD | PE-Cy7 conjugated anti-mouse NK1.1 antibody | 562062 | FACS |
| BD | APC conjugated anti-mouse NKG2A antibody | 564383 | FACS |
| Biolegend | PE-conjugated anti-mouse CD314 antibody | 115605 | FACS |
| BD | BV650 conjugated anti-mouse IFN-γ antibody | 563854 | FACS |
| BD | BV711 conjugated anti-mouse CD107a antibody | 564348 | FACS |
| Biolegend | BV605 conjugated anti-mouse PD-1 | 135220 | FACS |
| Biolegend | BV421 conjugated anti-mouse CTLA-4 | 106312 | FACS |
| Biolegend | BV510 conjugated anti-mouse TNF-α | 506339 | FACS |
| BD | anti-mouse Fc Block | 553141 | FACS |
| BD | Fixable Viability Stain 700 | 564997 | FACS |
| Abcam | Goat Anti-Mouse IgG H&L Alexa Fluor® 488 | ab150113 | IF |
| A32733TR | Goat Anti-Rabbit IgG H&L Alexa Fluor® Plus 647 | Invitrogen | IF |
| Bio cell | anti-mouse PD-1 | RMP1-14 | vivo assay |
| Selleckchem | anti-mouse CTLA-4 | A2103 | vivo assay |

**Supplement Table 2:** **Reagents involved in the manuscript**

| **Brand name** | **Reagents name** | **Cat No.** |
| --- | --- | --- |
| BD | Cytofix/Cytoperm™ Fixation/Permeabilization Solution Kit | 554714 |
| MCE | Ionomycin | HY-13434 |
| MCE | Phorbol 12-myristate 13-acetate (PMA) | HY-18739 |
| Biolegend | Brefeldin A | 420601 |
| MCE | MG132 | HY-13259 |
| Selleck | Cycloheximide | S7418 |
| Peprotech | Human TNF-α | 300-01A-10 |
| Peprotech | Human IFN-γ | 300-02-20 |
| Selleck | Nec-1s | S8641 |
| Selleck | GSK872 | S8465 |
| Selleck | Necrosulfonamide | S8251 |
| CSN pharm | BAY-11-7082 | CSN13487 |
| Beyotime | RIPA Lysis Buffer | P0013B, P0013 |
| Biomake | Protein A/G Magnetic-Beads | B23201 |
| Biomake | Protease Inhibitor Cocktail | B14001 |
| Biomake | Phosphatase Inhibitor Cocktail | B15001 |
| Thermo Scientific™ | Pierce BCA Protein Assay Kit | 23225 |
| Invitrogen | Puromycin | A11138-03 |
| 4ABiotech | UltraSignal ECL chemiluminescence substrate | 4AW011-100 |

**Supplement Table1: siRNA/shRNA targeting sequences involved in manuscript**

**Supplement Tables 3**

| Genes | siRNA Targeting sequences |
| --- | --- |
| Human-Casp8-1 | GTTCCTGAGCCTGGACTAC |
| Human-Casp8-2 | GCCCAAACTTCACAGCATT |
| Human-Casp8-3 | GATAATCAACGACTATGAA |
| Human-A20 | GGAAGAAATACACATATTT |
| Human-p65 | GGAGTACCCTGAGGCTATATT |
| Human-RIP1 | GCCAGCTGCTAAGTACCAATT |
| Mouse-Casp8-1 | GTTCCTGAGCCTGGACTAC |
| Mouse-A20 | CAA AGC ACT TAT TGA CAG A |

**Supplement Table2: PCR primers involved in manuscript**

**Supplement Table 4**

| Genes | Primers |
| --- | --- |
| Human-Casp8 | Forward:5’AGAGTCTGTGCCCAAATCAAC-3’ |
|  | Reverse:5’-GCTGCTTCTCTCTTTGCTGAA-3’ |
| Human-PD-L1 | Forward:5’TGTGGCATCCAAGATACAAACTCAAAG-3’ |
|  | Reverse: 5’-TCCTCCTCTGCTTTCGCCAGGTTC-3’ |
| Human-β-actin | Forward: 5’-GACTACCTCATGAAGATCCTCACC-3’ |
|  | Reverse: 5’-TCTCCTTAATGTCACGCACGATT-3’ |
| Casp8-C360S mutation | Forward:5’-TCCCAGGGGGATAACTACCAGAAAGGTATACCTG-3’ |
|  | Reverse: 5’-AGCCTGAATAAAAAACACTTTGGGT-3’ |
| A20-ZnF4 mutation | Forward: 5’-GCTGCTGGGACTCCAGAAAACAAGGGC-3’ |
|  | Reverse: 5’-CACGCAGCCGGCTTTTCTGCACTTG-3’ |
| A20-ZnF7 mutation | Forward: 5’-GCTAACGAAGCTTTTCAGTTCAAGCAG-3’ |
|  | Reverse: 5’-GTAGCCGTTGCACTTGGCATTGCCA-3’ |
